# Copper homeostasis and cuproptosis in tumor pathogenesis and therapeutic strategies

**DOI:** 10.3389/fphar.2023.1271613

**Published:** 2023-09-12

**Authors:** Chenbin Bian, Zhuangzhuang Zheng, Jing Su, Sitong Chang, Huiyuan Yu, Jindian Bao, Ying Xin, Xin Jiang

**Affiliations:** ^1^ Jilin Provincial Key Laboratory of Radiation Oncology and Therapy, The First Hospital of Jilin University, Changchun, China; ^2^ Department of Radiation Oncology, The First Hospital of Jilin University, Changchun, China; ^3^ NHC Key Laboratory of Radiobiology, School of Public Health of Jilin University, Changchun, China; ^4^ Key Laboratory of Pathobiology, Ministry of Education, Jilin University, Changchun, China

**Keywords:** copper homeostasis, cuproptosis, carcinogenesis, cancer therapeutics, drug

## Abstract

Copper is an indispensable micronutrient for the development and replication of all eukaryotes, and its redox properties are both harmful and beneficial to cells. An imbalance in copper homeostasis is thought to be involved in carcinogenesis. Importantly, cancer cell proliferation, angiogenesis, and metastasis cannot be separated from the effects of copper. Cuproposis is a copper-dependent form of cell death that differs from other existing modalities of regulatory cell death. The role of cuproptosis in the pathogenesis of the nervous and cardiovascular systems has been widely studied; however, its impact on malignant tumors is yet to be fully understood from a clinical perspective. Exploring signaling pathways related to cuproptosis will undoubtedly provide a new perspective for the development of anti-tumor drugs in the future. Here, we systematically review the systemic and cellular metabolic processes of copper and the regulatory mechanisms of cuproptosis in cancer. In addition, we discuss the possibility of targeting copper ion drugs to prolong the survival of cancer patients, with an emphasis on the most representative copper ionophores and chelators. We suggest that attention should be paid to the potential value of copper in the treatment of specific cancers.

## 1 Introduction

Cell proliferation is a fundamental physiological process essential for the development and homeostasis of multicellular organisms and leads to exponential tissue growth (Hanahan and Weinberg, 2011). Cell proliferation defects and/or abnormal elevations are the primary cause of injury, ageing and disease. A prime example of uncontrolled cellular proliferation is cancer; the survival of cancer cells and their proliferation, and engraftment in distant tissues are highly dependent on the ability of cancer cells to obtain adequate oxygen and nutrients in harsh environments (Ge et al., 2022). Cancer continues to be a primary health concern worldwide, as the number of cancer-related deaths and incidences of cancer are increasing annually. According to the 2022 World Cancer Report, 4.82 million new cancer cases and 3.21 million cancer-related deaths are estimated to occur annually in China, and, as a result, China ranks first in the world in terms of number of cases and deaths (Xia et al., 2022; Feng et al., 2023). Radiotherapy failure and poor tumor prognosis are primarily attributed to radioresistance.

As an essential micronutrient, copper is required for various signaling pathways and biological behaviors in almost all cell types (Vetlényi and Rácz, 2020). In recent years, multiple lines of evidence have indicated that copper is closely implicated in the cell proliferation and death pathways; in other words, excess copper can lead to cell death (Turski and Thiele, 2009; Tang et al., 2022). However, whether copper-induced toxicity is a novel type of programmed cell death remains controversial, and a clear picture of its mechanisms and specific forms has not yet emerged. In 2022, Tsvetkov and colleagues reported in the journal *Science* that intracellular copper accumulation triggers the oligomerization of mitochondrial lipoylated proteins and destabilizes Fe–S cluster proteins, leading to an independent cell death mode termed cuproptosis, distinct from apoptosis, necrosis, pyroptosis, or ferroptosis (Tsvetkov et al., 2022). The role of copper in tumor progression has long been a focus of research in the fields of cancer pathology and cell physiology, with a considerable number of researchers focusing on the crucial relationship between cuproptosis and cancer. Copper, a pro-angiogenic factor, activates tumor angiogenesis and metastasis (Xu et al., 2022). Chemoresistance and radioresistance are attributed to dysfunctional copper metabolism (Liu et al., 2022; Yang et al., 2022). Several studies have shown that elevated serum copper levels are associated with tumor stage and disease invasion in patients with colorectal, lung, and breast cancer (Baszuk et al., 2021; Cui et al., 2021; Tsang et al., 2022). In contrast, in malignant cells, cuproptosis interferes with lipid metabolism and contributes to oxidative stress, mitochondrial damage, and endothelial cell dysfunction (Halliwell and Chirico, 1993; Ruiz et al., 2021; Zhang et al., 2021). The administration of copper alone or in combination with ionophores disrupts cancer cell survival, making it possible to eliminate copper with chelators or supplement it with ionophores for anti-tumor clinical applications (Lu et al., 2022). This implies that additional investigations are needed to elucidate the precise roles of copper homeostasis and cuproptosis in tumorigenesis. Therefore, we review recent advances in the role of copper in cancer occurrence and progression from different perspectives. In addition, we discuss relevant copper-targeting potential strategies in pre-clinical and clinical trials for cancer therapy, provide key insights into valuable new clinical treatments for cuproptosis-related tumor manifestations, and highlight the most important challenges in this field.

## 2 Regulation of copper homeostasis in mammalian cells

Copper, as a kind of indispensable transition metal, is a double-edged: it is essential as a cofactor for enzymes across the mammalian kingdom, including Cu/Zn superoxide dismutase 1 (Cu/Zn-SOD), cytochrome c oxidase (CCO), lysyl oxidase (LOX), and ceruloplasmin (CP); however, even modest intracellular concentrations can cause metabolic dysfunction, resulting in biological death (Cobine et al., 2021; Garza et al., 2023). In the case of mammals, copper is obtained through consumption of certain foods, including nuts, organ meats, and seafood (Linder, 2020). Copper is mainly distributed in the muscle, liver, and brain in two oxidation states: cuprous (Cu^1+^) and cupric (Cu^2+^) (Chen et al., 2020). In biological systems, copper exists primarily in the Cu^2+^ form because Cu^1+^ is readily oxidized to Cu^2+^ in the presence of oxygen or other electron acceptors. Copper oxidation is reversible because Cu^2+^ can accept electrons from strong reductants, such as ascorbate and reduced glutathione (GSH) (Arredondo and Núñez, 2005).

**FIGURE 1:**
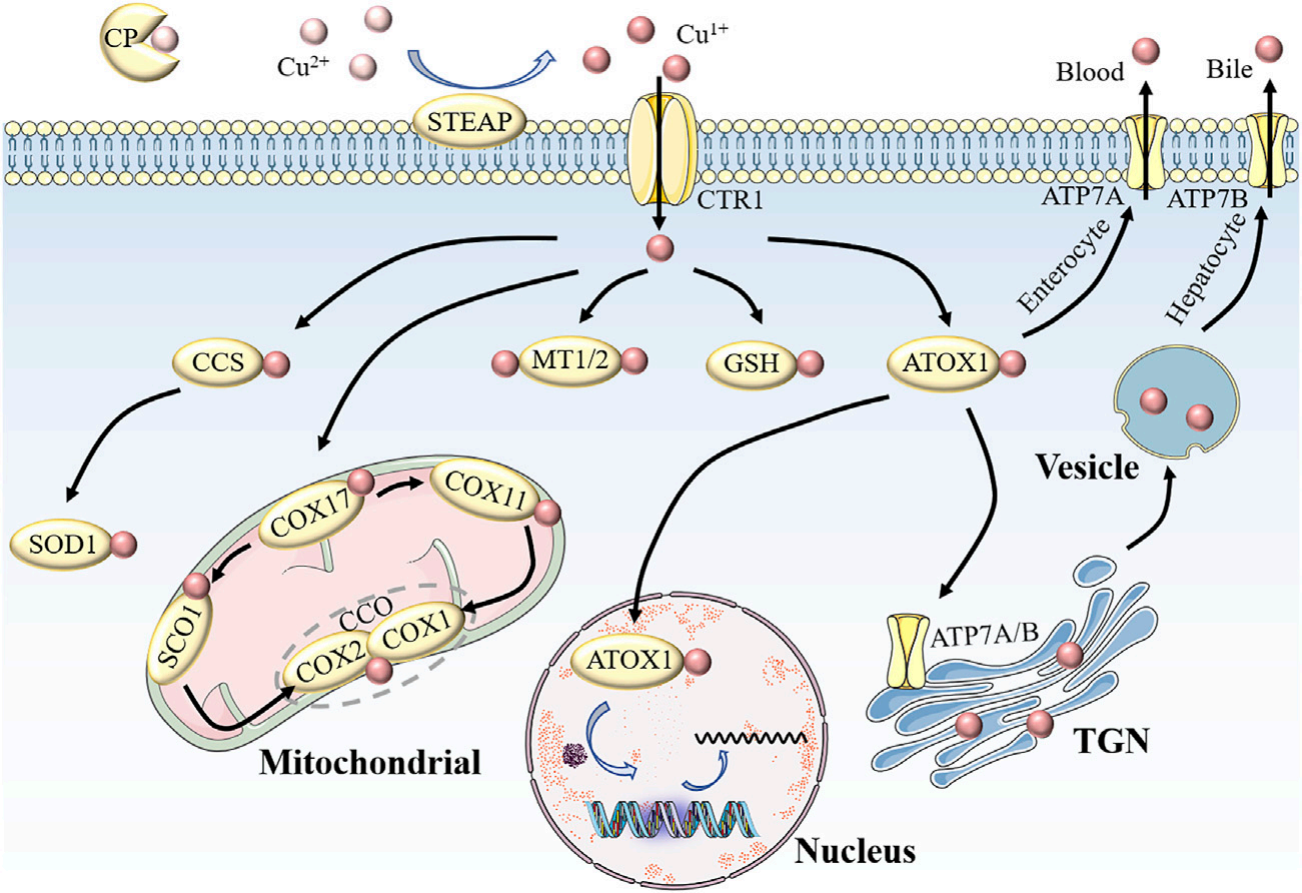
Schematic of copper homeostasis in mammalian cells.CP is the major protein carrier for exchangeable copper in blood plasma for circulation and delivery to organ and tissue systems. Extracellular Cu^2+^ is reduced to Cu^1+^ by STEAP, which in turn is transported into the cell by CTR1. Intracellular Cu^1+^ binds to different chaperone proteins to exert its unique functions. CCS delivers Cu^1+^ to SOD1 in the cytoplasm to clear free radicals. In the mitochondrial membrane space, COX17 transports Cu^1+^ to CCO to activate enzyme activity in the respiratory chain. MT1/2 and GSH are copper repositories that can bind Cu^1+^. Part of the Cu^1+^ carried by ATOX1 enters the nucleus to participate in gene expression, and the other part is pumped into the lumen of the TGN by ATP7A/B. When cytosolic Cu levels are high, Cu^1+^ in small intestinal cells is discharged into the portal circulation via ATP7A, while Cu^1+^ in liver cells is secreted into bile in the form of vesicles through ATP7B. The maintenance of cellular physiological functions is inseparable from copper homeostasis. Abbreviations: CP, ceruloplasmin; STEAP, the six-transmembrane epithelial antigen of the prostate; CTR1, copper transporter 1; CCS, copper chaperone for superoxide dismutase; SOD1, superoxide dismutase 1; MTI/2, metallothionein 1/2; GSH, glutathione; ATOX1, antioxidant 1 copper chaperone; ATP7A/B, ATPase copper transporter 7A/B; TGN, trans-Golgi network.

In mammals, copper homeostasis involves several key molecular targets (Figure 1). CP, albumin, and trans-copper proteins are the major protein carriers of exchangeable copper in blood plasma, resulting in delivery of copper to organs and tissues. Copper uptake occurs mainly in the small intestine, where epithelial cells take up copper ions via copper transporter 1 (CTR1) or solute carrier family 31 member 1 (SLC31A1), a transporter encoded by slc31a1 on the cell surface (Mandal et al., 2020). Because of the highly specific uptake of Cu^1+^ by CTR1, Cu^2+^ is reduced to Cu^1+^ by metallo-reductases, such as the six-transmembrane epithelial antigen of the prostate (STEAP), before entering the cells (Kleven et al., 2015). It is unclear which protein mediates copper absorption in the presence of CTR1 downregulation. The results of recent experiments suggest that the low-affinity copper transporter CTR2 may release copper from lysosomes or lysosome-like compartments for reutilization; that is, overexpression of CTR2 is associated with increased copper uptake (Prohaska, 2008).

Copper entering the cell binds to cytoplasmic or mitochondrial chaperone proteins, which, in turn, transfer copper to specific cellular destinations to perform its unique functions. Copper chaperone for superoxide dismutase (CCS), a cytosolic chaperone, plays a major role in oxidative stress (Miao and St Clair, 2009). The delivery of Cu to SOD1 requires the mediation of the CCS to detoxify reactive oxygen species (ROS) and maintain copper homeostasis. Studies have shown that mice with targeted disruption of CCS alleles experience significantly greater cell damage than controls, and this damage is likely caused by superoxide accumulation due to reduced SOD1 activity (Wong et al., 2000). In addition, SOD1 knockout mice are more likely to develop hepatocellular carcinoma, possibly due to oxidative damage to liver cells (Thadathil et al., 2022). However, SOD1 overexpression confers radioresistance in human glioma cells by suppressing irradiation-induced late ROS accumulation. These contradictory results may be due to the dual role of ROS in which the difference in ROS levels is dominant (Bian et al., 2022b). The copper chaperone for COX17, which is located in the cytoplasm and mitochondrial membrane space (IMS), is another copper metallochaperone involved in electron transfer in the oxidative respiratory chains (Lyons et al., 2012). In IMS, COX17 binds to and delivers Cu for either the synthesis of cytochrome oxidase 1 (SCO1) or COX11, which transfers Cu to CCO subunits (including COX1 and COX2), resulting in activation of enzymes in the mitochondrial respiratory complex (Nývltová et al., 2022). Therefore, we suggest that mutations in COX17, SCO1, and COX11 are associated with decreased CCO activity, and can be fatal. The third major copper chaperone protein is antioxidant-1 (ATOX1), which transfers copper from the trans-Golgi network (TGN) to copper-transporting ATPases (ATP7A and ATP7B) via the secretory pathway (Ash et al., 2021; Bitter et al., 2022). ATP7A and ATP7B exhibit different expression patterns in various tissues and organs. ATP7A in the basolateral membrane of enterocytes pumps copper into portal circulation and then into the liver, where excess copper ions are stored in the form of metallothionein 1 (MT1) and MT2 (Wang et al., 2023). Eventually, the copper in secretory vesicles is excreted into the bile via ATP7B on the bile canalicular membrane of hepatocytes, thus preventing the accumulation of copper (Wang et al., 2023). Dysregulation of copper metabolism is disadvantageous for cells because mutations in ATP7A and ATP7B are directly responsible for Menkes disease (MD) and Wilson disease (WD), respectively(Bitter et al., 2022). Children with MD exhibit severe symptoms, such as growth retardation, intellectual disability, neuronal degeneration, and connective tissue defects, which are associated with copper accumulation in intestinal cells as well as systemic copper deficiency, which is characterized by fulminant liver failure due to hepatic copper overload and copper accumulation-induced neuropsychiatric disorders in the brain (Chen et al., 2022). In addition, copper metabolism disorders are present in Alzheimer’s disease, atherosclerosis, and diabetes, and these findings undoubtedly further confirm the contribution of this metal to cellular pathophysiology (Mezzaroba et al., 2019; Philbert et al., 2022; Chen et al., 2023).

## 3 Cross-talk between components of cuproptosis and ferroptosis

All types of human cells inevitably self-destruct; cell death in response to unexpected stimulus signals is an uncontrolled biological process. Apoptosis, necroptosis, pyroptosis, and ferroptosis are tightly controlled modes of programmed cell death that play essential roles in development, tissue homeostasis, and defense against unwanted, redundant, and potentially dangerous cell growth (Bian et al., 2022a). Over the past few decades, there has been great interest in the connection between copper and regulated cell death, and the mechanism of copper-induced cell death has been extensively researched. Based on the findings of several well-known studies in the literature, it was erroneously believed that copper-dependent death is closely related to ROS and inflammation, and that it triggers oxidative stress-related cell death (Nagai et al., 2012; Yadav et al., 2013). However, it has been reported that cell death caused by copper overload was not reversed by using the 5 mM ROS inhibitor N-acetylcysteine (NAC), and the cytotoxic effect was only partially eliminated by 10 mM NAC; thus, copper may trigger a cell death pathway (Xie et al., 2023). Consistent with the experiments described above, Tsvetkov *et al.* also found that treatment with inhibitors of other known cell death mechanisms, including pan-caspase (Z-VAD-FMK and Boc-D-FMK), ferroptosis (ferrostatin-1), necroptosis (necrostatin-1), and oxidative stress (NAC), failed to abrogate copper ionophore–mediated cell death, and only copper chelators were able to prevent it, suggesting a mechanism distinct from that of previously identified cell death pathways (Tsvetkov et al., 2022).

Ferroptosis, a unique modality of iron-dependent cell death triggered by unrestricted lipid peroxides on cell membranes, plays an important role in various diseases, including cancer, neurodegeneration, and ischemic organ injury (Liang et al., 2022). Similarly, cuproptosis can be summarized as follows: excess Cu^2+^ within cells is transported to the mitochondria via copper ionophores (elesclomol); ferredoxin 1 (FDX1) reduces Cu^2+^ to Cu^1+^; lipoic acid synthetase (LIAS) converts the octanoylated domains into lipoylated derivatives; large amounts of Cu^1+^ bind directly to lipoylated components (including DBT, GCSH, DLST, and DLAT) of the tricarboxylic acid cycle, resulting in lipoylated proteins oligomerization and Fe-S cluster proteins loss, ensuing proteotoxic stress and, ultimately, cell death (Tong et al., 2022).

**FIGURE 2:**
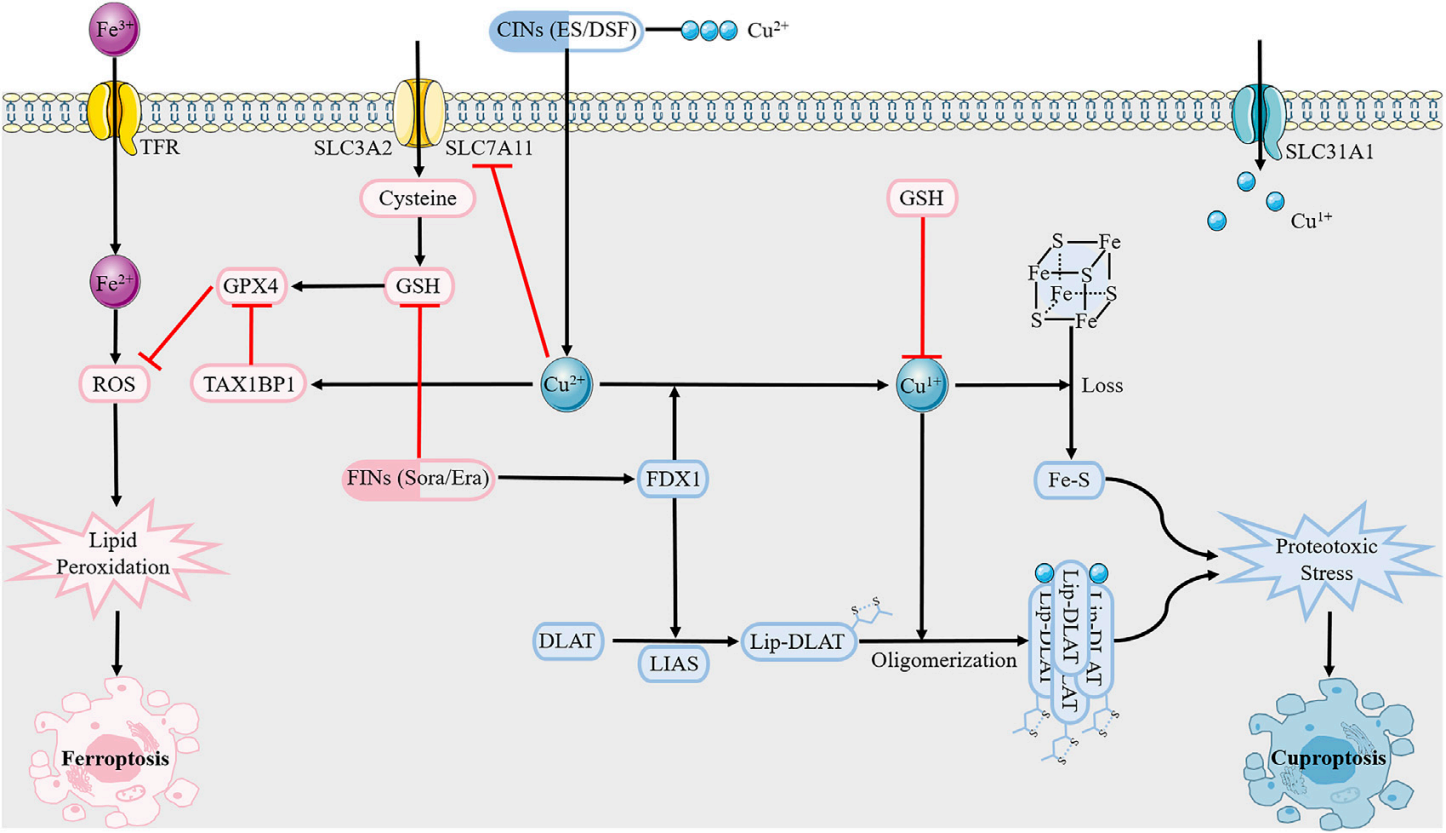
Overview of crosstalk between cuproptosis and ferroptosis.Cuproptosis inducers (CINs) elesclomol and disulfiram carry Cu^2+^ into the cell, which is reduced to Cu^1+^ under FDX1. Subsequent lipoylated proteins oligomerization and Fe-S cluster proteins loss trigger proteotoxic stress, and eventually cell death. As for ferroptosis, it is mediated by excess Fe^2+^ as well as abrogation of GSH biosynthesis and inactivation of GPX4 through causing lipid peroxidation. GSH, like copper chelator, inhibits copper death. However, ferroptosis inducers sorafenib and erastin can enhance cuproptosis, with potential mechanisms including inhibition of FDX1 degradation and reduction of GSH synthesis. Abbreviations: GSH, glutathione; GPX4, glutathione peroxidase 4; FDX1, ferredoxin 1; LIAS, lipoic acid synthetase; TFR, transferring receptor; SLC31A1, solute carrier family 31 member 1; SLC7A11, solute carrier family 7 member 11.

Although ferroptosis and cuproptosis are both metal-initiated modes of cell death, little is known about their interrelationship (Figure 2). Shen et al. (2022) *.* performed a comprehensive pan-cancer genomic analysis of the molecular correlations between cuproptosis and ferroptosis regulators in 33 cancer types, demonstrating crosstalk between the initiators, effectors, and executioners of cuproptosis and ferroptosis at the multiomic level. Exogenous copper increases ferroptosis sensitivity by inducing TAX1BP1-mediated autophagic degradation of glutathione peroxidase 4 (GPX4), independent of ROS generation, which is the theoretical basis for Cu^2+^-enhanced ferroptosis-mediated tumor inhibition in pancreatic cancer mouse models (Xue et al., 2023). The dithiocarbazate-copper complex synthesized by Xun *et al.* kills pancreatic cancer cells by triggering multiple mechanisms, including ferroptosis (Gou et al., 2021). In addition, amine oxidase copper-containing 1 (AOC1) exerts anti-cancer effects by acting on spermidine, leading to the activation of ROS and ferroptosis, which are significantly associated with reduced proliferation and migration of prostate cancer cells *in vitro* and *in vivo* (Ding et al., 2022). Notably, self-assembled copper-alanine nanoparticles (CACG) have great potential to enhance ferroptosis and immunotherapy for effective cancer treatment, as they help eliminate the extreme restriction of excessive GSH in the tumor microenvironment (TME) and low ROS generation efficiency (Song et al., 2023). This conclusion was further verified in the treatment of triple-negative breast cancer (TNBC) by nanoreactor Cu_2-x_Se (Li et al., 2023). A more recent study emphasized that copper can not only trigger iron-associated cell death but also activate caspases to cause apoptosis of liver cancer cells, which may provide a promising strategy to develop highly effective anti-tumor copper complexes (Cai et al., 2023). Disulfiram (DSF), a drug used to treat alcohol withdrawal syndrome, reacts with copper to form an anti-cancer metabolite (DSF/Cu) (Kannappan et al., 2021). DSF/Cu renders nasopharyngeal cancer cells or melanoma cells more vulnerable to ferroptosis by activating the ROS/MAPK and p53 signaling pathways or inhibiting the SLC7A11/GPX4 pathways, respectively, (Li et al., 2020; Li et al., 2023b). Interestingly, another chemical, elesclomol, causes copper overload within the mitochondria by promoting the degradation of ATP7A, leading to ROS accumulation, which further enhances oxidative stress and consequent ferroptosis in colorectal cancer cells (Gao et al., 2021). In contrast, Yang *et al.* confirmed that copper depletion induces ferroptosis. Overexpression of the copper metabolism MURR1 domain 10 (COMMD10) can reduce intracellular copper and disrupt the Cu-Fe balance to facilitate HIF1α degradation, resulting in impaired transcription of CP and SLC7A11, which jointly promote ferroptosis in hepatocellular cancer (HCC) cells(Yang et al., 2022). In addition to the bidirectional effect of copper on ferroptosis, ferroptosis inducers sorafenib and erastin also enhance cuproptosis in primary liver cancer cells by increasing copper-dependent lipoylated protein oligomerization, which is mediated by the inhibition of mitochondrial matrix-related protease-mediated FDX1 protein degradation and reduction of GSH synthesis (Wang et al., 2023). We should also not overlook other forms of cell death associated with cuproptosis. Copper induced autophagy through mtROS-dependent Akt/AMPK/mTOR signaling pathway, thereby protecting mouse monocytes from CuSO_4_-induced apoptosis (Luo et al., 2021). Copper-bacteriochlorin nanosheet, as a specific pyroptosis inducer, have been shown to enhance tumor immunogenicity and exert anti-tumor efficacy *in vivo* and *in vitro*, while minimizing systemic side effects (Zhang et al., 2023b). Given that cuproptosis is inextricably linked to apoptosis, ferroptosis, and pyroptosis, it is critical to further uncover the mechanisms of crosstalk between several modes of cell death. This highlights a new direction for the combined use of therapeutic drugs that target different modalities of cell death.

## 4 Mechanism of copper in carcinogenesis

**FIGURE 3:**
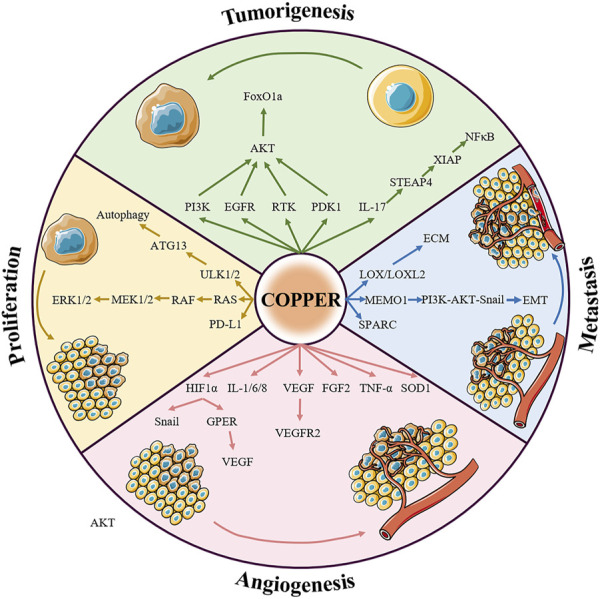
Summary of the relationship between copper signaling and cancer. Copper is involved in almost all fundamental processes of cancers. The pro-cancer role of copper can be summarized in four aspects: inducing tumorigenesis, promoting tumor growth, regulating angiogenesis, and assisting tumor metastasis, the details of which are presented below. Abbreviations: RTK, receptor tyrosine kinase; ERK1/2, extracellular signal-regulated kinase 1/2; ATK, agammaglobulinaemia tyrosine kinase; PI3K, phosphoinositide-3-kinase; PDK1, 3-phosphoinositide dependent protein kinase 1; FoxO1a, forkhead box O1a; LOX, lysyl oxidase; MEK1/2, mitogen-activated protein kinase kinase 1/2; MEMO1, mediator of ErbB2-driven cell motility 1; PD-L1, programmed death ligand 1; VEGF, vascular endothelial growth factor; GPER, G-protein estrogen receptor; FGF2, fibroblast growth factor 2; TNF-α, tumor necrosis factor α.

Given that copper is fundamental to cancer biology and a key factor in cell signaling, it is not surprising that it is gradually attracting much research interest; for example, studies on copper-induced cell death have been performed by cardiovascular disease and neurology teams. Copper directly binds to amyloid-β peptide, which is a pathological hallmark of Alzheimer’s disease, further increasing its aggregation and driving increased neurotoxicity (Cheignon et al., 2018). In addition, excess copper triggers Huntington’s disease by promoting the accumulation of Huntingtin proteins as well as inhibiting the activity of mitochondrial dehydrogenases (Mason et al., 2013; Xiao et al., 2013). Interestingly, high serum copper levels are associated with an increased risk of atherosclerotic disease, and conversely copper deficiency may contribute to hypertrophic cardiomyopathy (Dziedzic et al., 2022; Farrant et al., 2023). However, little is known about the mechanism of copper in carcinogenesis. Our review of the literature included statistical analyses that show that in individuals suffering from various malignancies, the concentration of copper in cancer tissues tends to be higher than that in the tissues of their origin, such as breast, thyroid, lung, gallbladder, pancreatic, and prostate cancer (Basu et al., 2013; Pavithra et al., 2015; Lener et al., 2016; Baltaci et al., 2017; Saleh et al., 2020; Wang et al., 2021). Copper is an indispensable cofactor in mitochondrial oxidative phosphorylation (OXPHOS), which provides the energy supply of malignant cells during rapid division (Tang et al., 2022). In addition to interfering with mitochondrial function, elevated copper levels affect glycolysis, lipid metabolism, insulin resistance, and the TME, which are integral to tumor cell proliferation, angiogenesis, distant metastasis, and drug insensitivity (Figure 3) (Wang et al., 2023).

The role of copper in promoting malignant cell growth and proliferation was discovered due to the critical role of the metal in receptor tyrosine kinase-related signaling pathways. The ion Cu^2+^ can activate receptor tyrosine kinases (RTK) without binding to the corresponding ligands, EGF and HGF. Activated RTK conducts upstream signaling to EGFR and MET, subsequently leading to the phosphorylation of downstream extracellular signal-regulated kinase (ERK) and agammaglobulinemia tyrosine kinase (ATK) (He et al., 2019). In addition, copper ions are also thought to activate downstream AKT by acting on phosphoinositide-3-kinase (PI3K) or 3-phosphoinositide dependent protein kinase 1 (PDK1) (Ostrakhovitch et al., 2002; Guo et al., 2021). Activation of AKT by copper can further lead to the phosphorylation and subcellular relocalization of the transcription factor forkhead box O1a (FoxO1a), ultimately leading to tumorigenesis (Walter et al., 2006). The mitogen-activated protein kinase (MAPK) pathway regulates tumor growth with the assistance of copper ions. Copper acts on mitogen-activated protein kinase 1 (MEK1) and enhances its ability to phosphorylate ERK1 and ERK2, thus stimulating RAF-MEK-ERK signaling (Baldari et al., 2019). For example, pharmacological inhibition of ATOX1 with the small molecule DCAC50 decreased the phosphorylation of ERK1/2 and reduced the growth of BRAF^V600E^-driven melanoma cell lines in a dose-dependent manner (Kim et al., 2019). Autophagy, as a dynamic degradation and recycling system, contributes to enhancing the resistance of cancer cells to stress (such as nutrient deprivation, hypoxia, DNA damage, metabolic stress, and chemotherapy) and sustains tumor metabolism and growth, ultimately driving carcinogenesis (Li et al., 2020). Copper is required to alleviate the inactivation of autophagic kinases ULK1 and ULK2 (ULK1/2) through direct Cu-ULK1/2 interactions. Genetic loss of Ctr1 or mutations in ULK1 that disrupt the binding of copper was found to reduce the growth of oncogene-driven lung adenocarcinomas (Tsang et al., 2020). Interestingly, the results of a recent study also showed that copper bridges the connection between chronic inflammation and tumor development; the authors showed that, in a murine model, the inflammatory response induces copper uptake through the IL-17-STEAP4-XIAP-NFκB axis to promote colon tumorigenesis (Liao et al., 2020).

Angiogenesis, the process by which new capillaries grow from preexisting blood vessels, is essential for the growth and metastasis of many solid tumors, including pancreatic, colorectal, and cervical cancer (Li et al., 2019; Wu et al., 2019; Zhang et al., 2023c). Hypoxia is an important microenvironmental factor that determines the rate of tumor angiogenesis, and the focus of the cell’s adaptation to hypoxia is the transcription factor hypoxia-inducible factor 1 α (HIF1α) (Paredes et al., 2021). Copper was originally found to have pro-angiogenic properties precisely because it can upregulate the expression of HIF1α (Xie and Kang, 2009). Li *et al.* demonstrated that copper deprivation significantly influenced breast cancer angiogenesis by inhibiting the HIF1α-Snail/Twist signaling pathway (Li et al., 2015). In particular, copper stabilizes nuclear HIF1α even under normoxic conditions, which in turn promotes the expression of vascular endothelial growth factor (VEGF) by cooperating with the G-protein estrogen receptor (GPER), leading to angiogenesis in breast and liver cancers (Martin et al., 2005; Rigiracciolo et al., 2015). SLC31A1 knockout endothelial cells exhibit reduced VEGF-induced VEGFR2 signaling, which is essential for developmental and reparative angiogenesis (Das et al., 2022). In addition, copper is implicated in the activation of many other pro-angiogenic factors, such as fibroblast growth factor 2 (FGF2), SOD1, tumor necrosis factor α (TNF-α), IL-1, IL-6, and IL-8 (Wang et al., 2023); for example, the number of blood vessels in tetrathiomolybdate-treated endometriosis-induced mice was much smaller than that in controls because copper depletion limits FGF2 mRNA expression (Delsouc et al., 2021).

Copper is an essential cofactor for various metalloenzymes with well-documented roles in tumor metastasis. As one of the classical secreted copper-dependent amine oxidases, members of the LOX family catalyze the crosslinking of elastin and collagen in the extracellular matrix, and are key mediators of tumor invasion (Leung et al., 2019). LOX/LOXL2 has been found to accelerate the spread of breast, colorectal, and prostate cancer (Baker et al., 2011; Cox et al., 2016). In breast cancer, not only is high expression of LOX related to bone metastasis, LOXL2 has also been shown to promote lung metastasis of breast cancer (Cox et al., 2015; Salvador et al., 2017). In an orthotopic mouse model of breast cancer, ATP7A silencing attenuated LOX activity and reduced the recruitment of myeloid cells to the lungs, thereby suppressing tumor metastasis (Shanbhag et al., 2019). In addition, the ATOX1-ATP7A-LOX axis is necessary for breast cancer cell migration, and high levels of ATOX1 often indicate poor patient survival (Blockhuys et al., 2020). In recent years, it has been gradually revealed that another copper-binding protein, the mediator of ErbB2-driven cell motility 1 (MEMO1), has a particularly relevant role in cancer cell metastasis. MEMO1 binds to insulin receptor substrate 1 (IRS1) and activates the downstream PI3K-Akt-Snail1 signaling pathway, thereby triggering the epithelial-mesenchymal transition program (Sorokin and Chen, 2013). ATOX1 was also found to interact with MEMO1 and exchange Cu^1+^ *in vitro* (Zhang et al., 2022). It is worth noting that SPARC (a collagen-binding glycoprotein) overexpression is closely related to increased aggressiveness of some cancers; however, the regulatory mechanism of copper still needs to be further explored (Morrissey et al., 2016).

Cancer immune evasion is recognized as a central hallmark of tumor development, and targeting programmed death receptor 1 (PD-1)/programmed death ligand 1 (PD-L1) to restore the normal anti-tumor immune response has been difficult (Mortezaee, 2020). A search of The Cancer Genome Atlas database showed that there is a positive correlation between CTR1 and PD-L1 expression in tumor tissues. Copper supplementation induces PD-L1 gene transcription and protein stabilization, whereas copper deprivation mediates the ubiquitination and degradation of PD-L1 through the downregulation of EGFR and STAT phosphorylation (Voli et al., 2020). Therefore, the repurposing of clinically available Cu chelators as immune checkpoint inhibitors may be a promising strategy. Elucidating the precise mechanism of copper in carcinogenesis would contribute to the individualized treatment of tumors. In the era of precision oncology, there is an urgent need to identify the molecular mechanisms underlying altered copper homeostasis in different types of cancer.

## 5 Cuproptosis and tumors

Cuproptosis can be regulated by specific cuproptosis-related genes (CRGs), including seven pro-cuproptosis genes (FDX1, LIAS, LIPT1, DLD, DLAT, PDHA1, and PDHB), three anti-cuproptosis genes (MTF1, GLS, and CDKN2A), and three key copper transporters: ATP7A, ATP7B, and SLC31A1 (Tsvetkov et al., 2022). An in-depth understanding of these CRGs in the context of cancer pathology is necessary to understand cuproptosis-related tumorigenesis and develop the cuproptosis pathway as a therapeutic target for cancer research. We outline the expression levels and clinical significance of CRGs in different tumors (Table 1).

**TABLE 1:** Functions and clinical values of cuproptosis-related genes.

Gene	Full name	Subcellular locations	Functions	Role in cuproptosis	Clinical value	Ref.
**FDX1**	Ferredoxin 1	Mitochondrion	Transfers electrons from NADPH through ferredoxin reductase to mitochondrial cytochrome P450, involved in steroid, vitamin D, and bile acid metabolism.	Ruduces Cu^2+^ to Cu^1+^	FDX1 high expression is associated with better prognosis in most tumors.	Ding et al. (2022a), Wang et al. (2022a), Xu et al. (2022a), Bian et al. (2022c), Wang et al. (2022c), Zhang et al. (2022c), Wang et al. (2023a)
**LIAS**	Lipoic Acid Synthetase	Mitochondrion	Catalyzes the conversion of the octanoylated domains to lipoylated derivatives.	Involved in lipoylation of DLAT	LIAS high expression is associated with better prognosis in STAD, KIRC, READ, BRCA, OV, and PADD, but on the contrary in LUAD.	Wang et al. (2022a), Cai et al. (2022), Huang et al. (2022)
**LIPT1**	Lipoyltransferase 1	Mitochondrion	Catalyzes the transfer of the lipoyl group from lipoyl-AMP to the specific lysine residue of lipoyl domains of lipoate-dependent enzymes.	Involved in lipoylation of DLAT	LIPT1 high expression is associated with better prognosis in SKCM, BLCA, PADD, BRCA, STAD, and OV, but on the contrary in UCEC, and LIHC.	Liu et al., 2022b; Chen (2022), Huang et al. (2022), Lv et al. (2022), Yan et al. (2022)
**DLD**	Dihydrolipoamide Dehydrogenase	Mitochondrion and nucleus	Component of the glycine cleavage system and E3 component of α-ketoacid dehydrogenase complexes.	Involved in lipoylation of DLAT	DLD high expression is associated with better prognosis in BRCA, and HCC, and indicates the pathological staging of LUAD.	Li et al. (2022b), Wang et al. (2022b), Jiang et al. (2022)
**DLAT**	Dihydrolipoamide S-Acetyltransferase	Mitochondrion	E2 component of the pyruvate dehydrogenase complex, catalyzes the overall conversion of pyruvate to acetyl-CoA and CO_2_.	Lipoylated DLAT oligomerization leads to cell death	DLAT high expression is associated with better prognosis in ccRCC, and CRC, but on the contrary in PADD, BRCA, LGG, and LIHC.	Li et al. (2022a), Bian et al. (2022c), Huang et al. (2022), Wu et al. (2022), Xu et al. (2023)
**PDHA1**	Pyruvate Dehydrogenase E1 Subunit Alpha 1	Mitochondrion and nucleus	E1 α1 component of the pyruvate dehydrogenase complex, catalyzes the overall conversion of pyruvate to acetyl-CoA and CO_2_.	Positively regulates cuproptosis	PDHA1 high expression is associated with better prognosis in KIRC, and CESC, but on the contrary in PRAD, LUAD, BRCA, and STAD.	Deng et al. (2022), Jiang et al. (2022), Cheng et al. (2023), Zhao et al. (2023)
**PDHB**	Pyruvate Dehydrogenase E1 Subunit Beta	Mitochondrion and nucleus	E1 β component of the pyruvate dehydrogenase complex, catalyzes the overall conversion of pyruvate to acetyl-CoA and CO_2_.	Positively regulates cuproptosis	PDHB high expression is associated with better prognosis in KIRC, and KIPR, and a lower stage in KIRP.	Zhang et al. (2023a)
**MTF1**	Metal Regulatory Transcription Factor 1	Nucleus	Induces expression of metallothioneins and other genes involved in metal homeostasis in response to heavy metals such as Cd, Zn, Cu, and Ag.	Negatively regulates cuproptosis	MTF1 high expression is associated with better prognosis in STAD, KIRC, LUNG, BRCA, and OV, but on the contrary in LIHC, and LGG.	Song et al. (2023a), He et al. (2023)
**GLS**	Glutaminase	Mitochondrion and cytosol	Catalyzes the hydrolysis of glutamine to glutamate and ammonia.	Negatively regulates cuproptosis	GLS high expression is associated with poorer prognosis in UCEC, PRAD, and HCC.	Zhang et al., 2022a; Chen (2022), Li et al. (2023a)
**CDKN2A**	Cyclin Dependent Kinase Inhibitor 2A	Mitochondrion, cytosol, and nucleus	Capable of inducing cell cycle arrest in G1 and G2 phases.	Negatively regulates cuproptosis	CDKN2A high expression is associated with poorer prognosis in ccRCC, UCEC, CRC, and BRCA, but on the contrary in HCC.	Ding et al. (2022a), Bian et al., 2022c; Chen (2022), Jiang et al. (2022), Wu et al. (2022)
**ATP7A**	ATPase Copper Transporting Alpha	Golgi apparatus, endosome, endoplasmic reticulum, plasma membrane, cytosol and nucleus	ATP-driven Cu^1+^ pump that plays an important role in intracellular copper ion homeostasis.	knock out leads to intracellular Cu^1+^ accumulation	ATP7A high expression is associated with poorer prognosis in HCC, and BRCA.	Li et al. (2022a), Li et al. (2022b)
**ATP7B**	ATPase Copper Transporting Beta	Golgi apparatus, endosome, plasma membrane and mitochondrion	ATP-driven Cu^1+^ pump that plays an important role in intracellular copper ion homeostasis.	knock out leads to intracellular Cu^1+^ accumulation	MTF1 high expression is associated with better prognosis in LGG, LUAD, BRCA, and HCC.	Li et al. (2022a), Bao et al. (2022), Li et al. (2022b), Zhu et al. (2023)
**SLC31A1**	Solute Carrier Family 31 Member 1	Plasma membrane	High-affinity, saturable copper transporter involved in dietary copper uptake.	Promotes intracellular Cu^1+^ accumulation	SLC31A1 high expression is associated with better prognosis in NSCLC, and HCC, but on the contrary in BRCA, ACC, MESO, and LGG.	Sun et al. (2018), Li et al. (2022a), Li et al. (2022b), Kong et al. (2023)

In clear cell renal cell carcinoma (ccRCC), high expression of FDX1 and DLAT predicts better survival; however, CDKN2A exhibits carcinogenic features, the overexpression of which is associated with worse survival in patients with ccRCC (Bian et al., 2022). This may be because FDX1 and CDKN2A are involved in the regulation of immune cell infiltration in pantumors (Chen et al., 2021). Furthermore, compared to paired normal tissues, the expression levels of most CRGs were upregulated in low-grade gliomas (LGG), in addition to ATP7B. A high CRG score implied higher TME scores, more significant TME cell infiltration, and an increased mutation burden. Their study showed that the potential effects of CRGs on the TME and chemoradiotherapy sensitivity are independent predictors of prognosis in patients with LGG (Bao et al., 2022). Sha *et al.* performed a comprehensive analysis of CRGs in 346 TNBC specimens. Groups with high expression of ATP1A, DLST, and LIAS are characterized by high tumor mutation burden and immune activation, good survival probability, and greater immunoreactivity to cytotoxic T lymphocyte antigen 4 (CTLA4), whereas groups with high expression of LIPT1 and PDHA1 are characterized by the activation of stromal pathways and immunosuppression (Sha et al., 2022). These results provide new targets for the development of novel anti-cancer drugs. Pancreatic adenocarcinoma (PAAD) is a highly malignant tumor with a 5-year overall survival rate of less than 10%. Polygenic prognostic studies based on cuproptosis may overcome barriers that have stalled the development of treatments. Currently, three essential CRGs (DLAT, LIPT1, and LIAS) have been identified as potential diagnostic biomarkers (Huang et al., 2022). FDX1 was significantly downregulated in HCC, and a cuproptosis-related risk score (CRRS) based on FDX1 and its associated genes was constructed using the LASSO Cox regression model. The high-CRRS group showed a lower OS, which may be attributed to a high mutational frequency of tumor suppressor genes such as tumor protein P53 (TP53) and breast cancer susceptibility gene 1 (BRCA1)-associated protein 1 (BAP1) in high-CRRS HCC patients (Zhang et al., 2022). Lipoyltransferase 1, encoded by LIPT1, is involved in lipoic acid metabolism, and LIPT1 silencing inhibits the tricarboxylic acid cycle. Similarly, high LIPT1 expression in skin cutaneous melanoma (SKCM) and bladder cancer (BLCA) has been suggested to improve prognosis (Chen et al., 2021). Moreover, LIPT1 expression is positively correlated with PD-L1 expression and negatively associated with Treg cell infiltration, suggesting that LIPT1 can guide immunotherapy in patients with cancer (Lv et al., 2022). Although previous research on CRGs has revealed the ways in which they may influence or be influenced by cuproptosis as well as the potential significance of their involvement in the connection between cuproptosis and cancers, additional clinical testing of novel therapies based on this principle are required in order to verify the clinical indications and safety.

## 6 Therapeutic strategies for targeting copper in cancer

Chemotherapy is the main treatment for malignant tumors, and the emergence of new targeted drugs has changed the tumor treatment model and opened up an era of precision medicine. Through numerous clinical practices, it has been shown that targeted therapy can not only selectively intervene in the molecules and pathways involved in tumor growth and development but also reduce the risk of tumor progression, thereby prolonging patient survival (Pérez-Herrero and Fernández-Medarde, 2015). Given the central role of copper in tumorigenesis, recent years have witnessed an explosion of interest in developing therapeutic strategies that leverage copper-dependent disease responses. Copper chelators that inhibit cuproplasia and copper ionophores that promote cuproptosis have shown great potential for cancer-targeted therapy (Table 2).

**TABLE 2:** Copper-targeting agents in clinical trials for cancer treatment.

Agents	Role	Identifier	Combination	Status	Phase	Cancer type
**Tetrathiomolybdate**	Chelator	NCT00150995	N.A.	Completed	II	Hormone Refractory Prostate Cancer
		NCT01837329	Carboplatin/​Pemetrexed	Completed	I	Metastatic Non-small Cell Lung Cancer
		NCT00195091	N.A.	Active	II	Breast Cancer
		NCT00006332	N.A.	Completed	II	Hepatocellular Carcinoma
		NCT00176800	Chemoradiation	Completed	II	Esophageal Carcinoma
		NCT00560495	Radiation Therapy	Withdrawn	I	Stage I/II/III Non-Small Cell Lung Cancer
		NCT00176774	Irinotecan/Leucovorin/5-Fluorouracil	Completed	II	Colorectal Carcinoma
**ATN-224**	Chelator	NCT00405574	N.A.	Unknown	II	Prostate Cancer
		NCT00383851	Temozolomide	Unknown	II	Advanced Melanoma
		NCT00352742	Bortezomib	Terminated	I/II	Multiple Myeloma
		NCT00674557	Exemestane	Terminated	II	Recurrent or Advanced Breast Cancer
**Trientine**	Chelator	NCT03480750	Pegylated Liposomal Doxorubicin/Carboplatin	Completed	I/II	Epithelial Ovarian Cancer
		NCT01178112	Carboplatin	Completed	I	Advanced Malignancies
		NCT02068079	Vemurafenib	Withdrawn	I	BRAF Mutated Metastatic Melanoma
**Penicillamine**	Chelator	NCT00003751	Low Copper Diet/Radiation Therapy	Completed	II	Glioblastoma
**Elesclomol**	Ionophore	NCT00522834	Paclitaxel	Terminated	III	Melanoma
		NCT01280786	N.A.	Unknown	I	Relapsed or Refractory Acute Myeloid Leukemia
		NCT00827203	N.A.	Suspended	I	Solid Tumors
		NCT00808418	Docetaxel/Prednisone	Completed	I	Metastatic Prostate Cancer
		NCT00888615	Paclitaxel	Completed	II	Recurrent or Persistent Ovarian Epithelial Cancer, Fallopian Tube Cancer, or Primary Peritoneal Cancer
		NCT00087997	Paclitaxel	Completed	II	Soft Tissue Sarcomas
		NCT00084214	Paclitaxel	Completed	I/II	Melanoma
		NCT00088114	Paclitaxel	Completed	I	Solid Tumors
		NCT00088088	Paclitaxel/Carboplatin	Completed	I/II	Stage IIIB/​IV Non-Small Cell Lung Cancer
**Disulfiram**	Ionophore	NCT03323346	Copper	Recruiting	II	Metastatic Breast Cancer
		NCT02678975	Copper	Completed	II/III	Recurrent Glioblastoma
		NCT03950830	Cisplatin	Unknown	II	Refractory Germ Cell Tumors
		NCT05667415	Cisplatin	Not Yet Recruiting	N.A.	Advance Gastric Cancer
		NCT01118741	N.A.	Completed	N.A.	Recurrent Prostate Cancer
		NCT00256230	N.A.	Completed	I/II	Metastatic Melanoma
		NCT03151772	Bioavailability	Terminated	I	Glioblastomas
		NCT02101008	Chelated Zinc	Completed	II	Refractory Disseminated Malignant Melanoma
		NCT02671890	Gemcitabine Hydrochloride	Active	I	Refractory Solid Tumors or Metastatic Pancreatic Cancer
		NCT00312819	Chemotherapy	Completed	II/III	Lung Cancer
		NCT05210374	Copper Gluconate/Liposomal Doxorubicin	Recruiting	I	Treatment-Refractory Sarcomas
		NCT04521335	Copper Gluconate	Terminated	I	Treatment-Refractory Multiple Myeloma
		NCT04265274	Vinorelbine/Cisplatin/Copper	Unknown	II	CTC_EMT Positive Refractory Metastatic
		NCT03714555	Paclitaxel/Gemcitabine	Completed	II	Metastatic Pancreatic Cancer
		NCT02715609	Radiation Therapy/Temozolomide	Active	I//I	Newly Diagnosed Glioblastoma
**Clioquinol**	Ionophore	NCT00963495	N.A.	Terminated	I	Relapsed or Refractory Hematological Malignancy

Copper chelators were initially designed to treat MD/WD but have not been evaluated as antitumor agents in recent years. To date, copper chelators have been used in several clinical trials against copper-overloaded tumors. The earliest available drug is tetrathiomolybdate, which inhibits lung metastasis of head and neck tumors and breast cancer by reducing LOX activity (Kumar et al., 2010; Chan et al., 2017). ATN-224, a second-generation analog of ammonium tetrathiomolybdate, also showed potent anti-tumor effects. Researchers have found that ATN-224 has the dual ability to degrade SOD1 and CCO, which is devastating for the survival of patients with diffuse large B-cell lymphoma (Lee et al., 2013). Importantly, copper chelators can be repurposed as adjuvants in conventional cancer therapy to reverse the insensitivity of some tumors to chemoradiotherapy. The best example is D-penicillamine, which can inhibit tumor growth in oxaliplatin-resistant human cervical cancer cells by interfering with the Sp1-hCtr1-p53-ATP7A axis and enhancing the lethality of radiation and carboplatin against lung and breast cancer cells (Chen et al., 2015; Sciegienka et al., 2017). Synergy with immune checkpoint inhibitors is another significant finding of copper chelators. Florida *et al.* confirmed that copper chelators mediated the ubiquitination degradation of PD-L1, promoted an increase in tumor-infiltrating CD4^+^ and CD8^+^ lymphocytes, and activated Natural Killer cells in a glioblastoma mouse model(Florida et al., 2019). However, the toxicity of copper chelators cannot be ignored because they deplete copper throughout the body in a nonselective manner. Mitochondria-targeted copper-depleting nanoparticles (CDNs) deprive cancer cells of copper in the mitochondria, resulting in a metabolic switch to glycolysis by decreasing oxygen consumption and OXPHOS, and ultimately suppressing TNBC in mice (Cui et al., 2021).

Copper ionophores, also known as copper proptosis-related drugs, can improve the bioavailability of copper in cells. Typical copper ionophores, DSF and elesclomol, confer the characteristic ability to transfer copper ions from the extracellular to the intracellular space, subsequently triggering excess ROS-mediated tumor cell death (Ge et al., 2022). Elesclomol, a copper ionophore that targets mitochondrial metabolism in cancer therapy, shows significant inhibitory effects on cancer stem cells, drug-resistant cancer cells, and cells with lower glycolytic activity (Zheng et al., 2022). A randomized, double-blind, phase II clinical trial showed that the addition of elesclomol to paclitaxel for the treatment of stage IV metastatic melanoma significantly doubled the median PFS, with an acceptable toxicity profile and prolonged OS (O'Day et al., 2009). Although elesclomol and paclitaxel combination therapy did not achieve the PFS endpoint in a subsequent Phase III study, a prospectively defined subgroup analysis revealed statistically significant improvement in patients with normal baseline levels of lactate dehydrogenase (LDH) (O'Day et al., 2013). This is due to the high mitochondrial metabolism in patients with low serum LDH levels; in other words, serum LDH levels correlate with esclomole sensitivity. Notably, esclomole, while inducing cuproptosis in a mouse model of subcutaneous bladder cancer, was found to bind to an anti-programmed cell death protein ligand-1 antibody (αPD-L1), resulting in enhanced cancer immunotherapy (Guo et al., 2023). The serendipitous discovery of the anticancer effects of DSF can be traced back to 1977, when its chemosensitizing effects were demonstrated. The mechanisms by which DSF combined with Cu reverses cancer drug resistance include the suppression of ALDH, inhibition of NF-κB, activation of the MAPK pathway, inhibition of the ubiquitin-proteasome pathway, and remodeling of the tumor immune microenvironment (Li H. et al., 2020). In addition, targeting the p97-NPL4-UFD1 axis is one of the mechanisms by which DSF exerts its anti-tumor effects (Skrott et al., 2017). Unfortunately, few clinical trials of DSF have achieved the expected results owing to the inefficient delivery of DSF and Cu^2+^ to tumor sites and small sample sizes. For example, a phase II/III clinical trial found that among patients with recurrent glioblastoma, DSF combined with temozolomide led to significantly increased toxic effects but no significant difference in survival, compared with chemotherapy alone, suggesting that DSF and copper did not benefit patients with recurrent glioblastoma (Werlenius et al., 2023). Taken together, these findings suggest that altering the intracellular copper concentration could be a promising therapeutic strategy for a subset of tumors. Indeed, as the therapeutic index is the decisive factor for the utility of any therapy, those targeting copper are often limited by side effects rather than a lack of efficacy. Therefore, there is an urgent need to validate the most appropriate drug dosage through preclinical and clinical trials, especially in cancer patients without abnormal copper metabolism.

## 7 Conclusion and perspectives

Copper is a cofactor for enzymes involved in crucial metabolic steps and regulates cell proliferation, angiogenesis, metastasis, and drug resistance in cancers. Both deficiency and overload of intracellular copper can negatively affect the human body. In normal cells, the maintenance of copper homeostasis depends on stable copper metabolism. A moderate increase in copper concentration establishes the chronic oxidative stress environment required for cancer growth, known as cuproplasia. If copper levels continue to increase beyond the antioxidant capacity of the cells, cancer cells will be forced to undergo cuproptosis. Therefore, the targeting of copper ions to inhibit tumorigenesis has received considerable attention. However, the use of copper chelators or ionophores alone has not shown any clinical benefits. In addition, owing to the lack of high specificity, the indiscriminate attack of drugs on non-tumor cells can have unwanted effects, which hinders their generalization. Exploring specific metabolic processes or molecules in different types of tumors may provide an important reference for optimizing drug treatment using copper ions. Currently, the field of cuproptosis is nascent in many ways. The lack of reliable cuproptosis biomarkers and the absence of further randomized clinical trials to confirm a direct relationship between cuproptosis and cancer are long-term bottlenecks limiting the promotion of cuproptosis in clinical applications. Despite these challenges, with a deeper understanding of the role of cuproptosis in various pathophysiological conditions, a breakthrough in applying cuproptosis to treat or prevent copper-related diseases is just over the horizon, and thus deserves renewed attention.

## Figures and Tables

**Table:** 

**AOC1**	Amine oxidase copper-containing 1
**ATK**	Agammaglobulinaemia tyrosine kinase
**ATOX1**	Antioxidant-1
**ATP7A/B**	Copper-transporting ATPase A/B
**CACG**	Copper-alanine nanoparticles
**CCO**	Cytochrome c oxidase
**CCS**	Superoxide dismutase
**COMMD10**	Copper metabolism MURR1 domain 10
**CP**	Ceruloplasmin
**CRGs**	Cuproptosis-related genes
**CTLA4**	Cytotoxic T lymphocyte antigen 4
**CTR1**	Copper transporter 1
**Cu/Zn-SOD**	Cu/Zn superoxide dismutase 1
**DSF**	Disulfiram
**ERK**	Extracellular signal-regulated kinase
**FDX1**	Ferredoxin 1
**FGF2**	Fibroblast growth factor 2
**FoxO1a**	Forkhead box O1a
**GPER**	G-protein estrogen receptor
**GPX4**	Glutathione peroxidase 4
**GSH**	Glutathione
**HIF1α**	Hypoxia-inducible factor 1 alpha
**IRS1**	Insulin receptor substrate 1
**LDH**	Lactate dehydrogenase
**LIAS**	Lipoic acid synthetase
**LOX**	Lysyl oxidase
**MAPK**	Mitogen-activated protein kinase
**MD**	Menkes disease
**MEK1**	Mitogen-activated protein kinase kinase 1
**MEMO1**	Mediator of ErbB2-driven cell motility 1
**MT1**	Metallothionein 1
**PD-1**	Programmed death receptor 1
**PDK1**	3-phosphoinositide dependent protein kinase 1
**PD-L1**	Programmed death ligand 1
**PI3K**	Phosphoinositide-3-kinase
**ROS**	Reactive oxygen species
**RTK**	Receptor tyrosine kinase
**SLC31A1**	Solute carrier family 31 member 1
**STEAP**	Six-transmembrane epithelial antigen of the prostate
**TGN**	Trans-Golgi network
**TNF-α**	Tumor necrosis factor α
**VEGF**	Vascular endothelial growth factor
**WD**	Wilson disease
